# Correlation Analysis of Lignin Accumulation and Expression of Key Genes Involved in Lignin Biosynthesis of Ramie (*Boehmeria nivea*)

**DOI:** 10.3390/genes10050389

**Published:** 2019-05-22

**Authors:** Yinghong Tang, Fang Liu, Hucheng Xing, Kaiquan Mao, Guo Chen, Qingquan Guo, Jianrong Chen

**Affiliations:** 1 College of Life and Environmental Sciences, Hunan University of Arts and Science, Changde 415000, Hunan, China; tyhisw1314@163.com; 2 College of Biological and Environmental Engineering, Changsha University, Changsha 410003, Hunan, China; lf800825@163.com (F.L.); 18007399571@163.com (K.M.); guochen9797@163.com (G.C.); qingquan_guo@126.com (Q.G.); 3 College of Agriculture, Hunan Agricultural University, Changsha 410128, Hunan, China; xhcsoldier@163.com

**Keywords:** ramie (*Boehmeria nivea*), lignin, key gene expression, correlation analysis, enzyme activity assay

## Abstract

The phloem of the stem of ramie (*Boehmeria nivea*) is an important source of natural fiber for the textile industry. However, the lignin content in the phloem affects the quality of ramie phloem fiber. In this study, the lignin content and related key gene expression levels were analyzed in the phloem and xylem at different developmental periods. The results showed that the relative expression levels of lignin synthesis-related key genes in the xylem and phloem of the stem gradually decreased from the fast-growing period to the late maturation period, but the corresponding lignin content increased significantly. However, the relative expression levels of a few genes were the highest during the maturation period. During all three periods, the lignin content in ramie stems was positively correlated with the expression of genes, including *PAL*, *C4H* and *4CL1* in the phenylpropanoid pathway, *F5H* and *CCoAOMT* in the lignin-specific synthetic pathway, and *CAD* in the downstream pathway of lignin synthesis, but the lignin content was negatively correlated with the expression of genes including *4CL3* in the phenylpropanoid pathway and *UDP-GT* in the shunt pathway of lignin monomer synthesis. The ramie 4CL3 recombinant protein prefers cinnamic acid as a substrate during catalysis, and it negatively regulates lignin synthesis. It is speculated that ramie *4CL3* is mainly involved in the synthesis of ramie flavonoid compounds, and that *4CL1* is mainly involved in lignin synthesis.

## 1. Introduction

Ramie (*Boehmeria nivea* L.), a subshrub or shrub species belonging to the family Urticaceae and the genus *Boehmeria* Jacq., is one of the most widely cropped herbaceous plants in southern China. It has been expanded to many uses [1,2,3,4], but it is mainly used in the textile industry. The stem of ramie can be divided into xylem and phloem, the phloem fiber can be used in industry. Among various bast fibers, ramie phloem fiber is the longest and shows the most strength, and ramie phloem fiber is two to seven times longer than the best cotton fiber; its thickness is two to three times greater than that of flax and hemp; and it is 20% lighter than cotton of the same volume. Textiles made using ramie phloem fiber as the raw material have a high lignin content and other noncellulose components; the structure of ramie phloem fiber is large and coarse, and their textile properties are not ideal. Therefore, textiles from ramie phloem fiber cannot be mass produced, and high fiber rigidity also causes the fabric to have an itchy sensation on the skin [5]. Lignin is an important macromolecular substance in plants and is widely present in vascular plants [6]. Lignin, however, may even be produced in the closest relatives to land plants: several studies have found lignin-like components in streptophyte algae and the genes in the phenylpropanoid and lignin biosynthesis pathway are present in those algae [7,8,9,10,11]. In the textile industry, lignin in phloem is considered to be an important factor that causes the pollution of fiber processing waste and affects fiber quality. Therefore, to overcome the problems caused by lignin accumulation in ramie, the correlation between the expression of key lignin synthesis-related genes and lignin accumulation in xylem and phloem of ramie should be elucidated; the results would provide a theoretical basis for the use of genetic engineering to develop ramie plants that produce environmentally friendly ramie phloem fiber with a low lignin content. Appropriate reduction of lignin content in phloem of ramie by genetic engineering will not have other negative effects on plant cultivation such as an enhanced infection rate [12,13].

Lignin is a phenolic polymer aggregated from monomers formed during the shikimate pathway, phenylpropanoid pathway, and lignin synthetic pathway. Current research on the lignin synthetic pathway mainly focuses on three aspects: first, the structural genes in the phenylpropanoid pathway, the expression levels of which affect lignin synthesis and content; these genes include phenylalanine alanine ammonia-lyase (*PAL*), cinnamate-4-hydroxylase (*C4H*), coumarate-3-hydroxylase (*C3H*), and 4-coumarate coenzyme A ligase (*4CL*); second, genes in the lignin-specific synthetic pathway, the expression of which greatly influences the composition of lignin monomers; these genes include caffeic acid-O-methyltransferase (*COMT*), coenzyme-AO-methyltransferase (*CCoAOMT*), and ferulate-5-hydroxylase (*F5H*); third, genes in the downstream pathways of lignin synthesis, which are mainly responsible for the reduction of monomers and greatly affect the synthesis of lignin monomers, these genes include cinnamoyl-CoA reductase (*CCR*), cinnamyl alcohol dehydrogenase (*CAD*), peroxidase (*POD*), and three other genes [14].

Studies have found that key genes for lignin accumulation in many plants have been proved, such as *CCoAOMT*, *4CL*, *COMT*, and *F5H* are the key genes in the xylem of *Linum usitatissimum* [15]; *PAL*, *4CL*, *C4H*, *CAD*, *CCoAOMT*, and *CCR* are the key genes for lignin accumulation in common buckwheat stems [16]; and *PAL*, *4CL*, *F5H*, and *CCR* are the key genes for lignin accumulation in the rape stem [17]. At present, some key genes in the lignin synthetic pathway of ramie have been reported, such as *CCoAOMT* [18], *COMT* [19], *4CL* [20], *CCR* [21], *CAD* [22], and *PAL* [23]. However, the expression of the key genes in the lignin synthetic pathway in ramie stems and their correlation with lignin accumulation have not been analyzed.

In recent years, research has been conducted to identify the key genes responsible for lignin synthesis, and studies have focused on reducing the lignin content and changing the lignin components by regulating the expression of genes such as *COMT*, *4CL*, and *C3H* through genetic engineering mainly in model plants such as tobacco [24], *Arabidopsis thaliana* [25], and the xylem-based woody plant poplar [26]. The lignin content in the phloem of stems is closely related to ramie phloem fiber in terms of its quality. Therefore, an evaluation of lignin synthesis and its regulation, as well as the functions of related genes responsible for lignin synthesis in ramie, is crucial.

**Figure 1:**
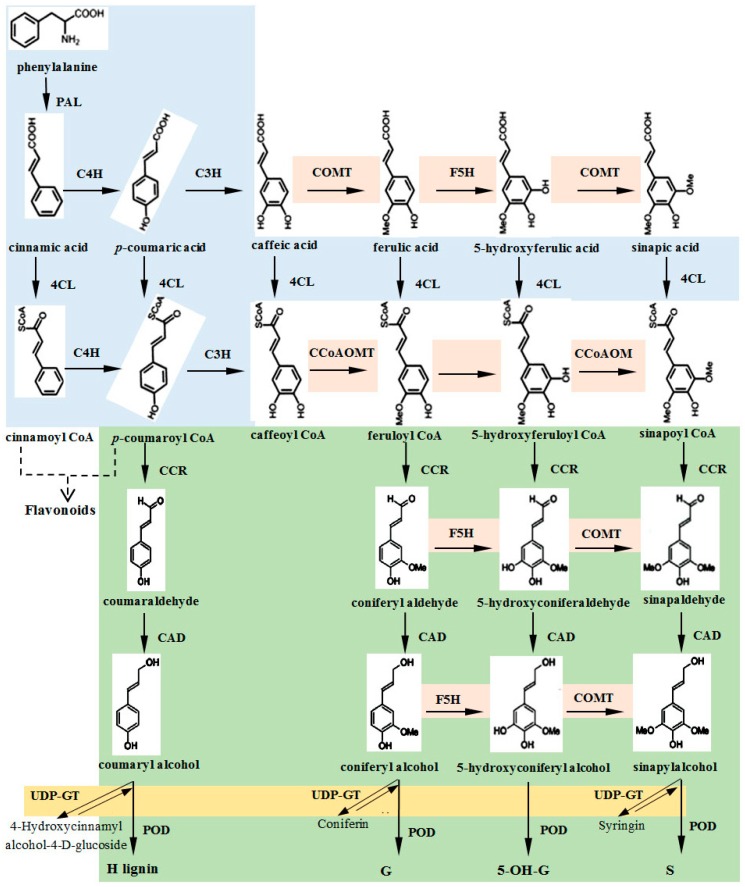
Phenylpropanoid pathway leading to lignin synthesis was obtained from the KEGG database of ramie [27]. Blue indicates the phenylpropanoid pathway; orange indicates the lignin-specific synthetic pathway; green indicates the downstream pathway of lignin synthesis; gold indicates the shunt pathway of lignin monomer synthesis.

In this study, the stems of the ramie cultivar Xiangzhu No. 3 were used as the study material; the lignin content in the stems during three periods (fast growing period, maturation period, and late maturation period) and at two sites (xylem and phloem) was determined through ultraviolet spectrophotometry. The key genes involved in the four pathways of lignin synthesis were identified based on registered data in GenBank and the ramie transcriptome [27] (Figure 1), including *PAL*, *C4H*, *C3H*, and *4CL* (including *4CL1* and *4CL3*) in the phenylpropanoid pathway; *COMT*, *F5H*, and *CCoAOMT* in the lignin-specific synthetic pathway; *CCR*, *CAD*, and *POD* in the downstream pathway of lignin synthesis; and coniferyle alcohol glucosyltransferase (*UDP-GT*) in the shunt pathway of lignin monomer synthesis. Quantitative real-time PCR (qRT-PCR) was used to analyze the expression level of these 12 genes and their correlation with the lignin content. The critical period in which lignin accumulation occurs in the xylem and phloem of ramie stems was explored, and the key genes for lignin accumulation in the stem were investigated. Moreover, the recombinant protein of the key enzyme 4CL3 was expressed in *Escherichia coli* M15, and its enzymatic activity was analyzed to explore the influence of the aforementioned genes on the flow direction (upstream and downstream) of substances (five substances include *p*-coumaric acid, cinnamic acid, caffeic acid, ferulic acid and sinapinic acid). The results of this study can provide insights into the development mechanism of the ramie stem; an understanding of the mechanism will improve the quality and yield of ramie phloem fiber, and it will also provide a foundation for the cultivation of environmentally friendly ramie plants having phloem with low lignin content.

## 2. Materials and Methods

### 2.1. Sample Preparation

The ramie cultivar Xiangzhu No. 3 was planted at the Department of Biological and Environmental Engineering of Changsha College. The stems in the fast-growing period (20 days after emergence), maturation period (40 days after emergence), and late maturation period (full bloom stage; 60 days after emergence) were selected as the study materials. Random selection of 6 plants with the same growth tendency were cut into small pieces of 1 cm. For these pieces, the bark (phloem) and the stem (xylem) of the stem section were separated, and quickly frozen in liquid nitrogen. After mixing 6 materials, they were evenly stored in a refrigerator at −80 °C for the extraction of total RNA, and the remaining sample materials were dried in an oven at 60 °C and ground to a powder in a tissue grinding mill MM 400 (RETSCH, Haan, Germany). The powder was sieved through a 20-mesh screen to determine the lignin content.

### 2.2. Total RNA Extraction and Integrity Testing

Total RNA from ramie stems was extracted using an RNA prep Pure Plant Plus Kit (TIANGEN, Beijing, China). The total RNA concentration was then measured using a Thermo Fisher Scientific Oy spectrophotometer 1510 microplate reader, and the integrity of the total RNA was determined through 1.0% agarose gel electrophoresis. A FastQuant RT Kit (TIANGEN, Beijing, China) was used to reverse transcribe the extracted total RNA to cDNA. After the quality of cDNA was tested using primers for the reference gene *Actin I* and the cDNA concentration was determined, the samples were stored at −20 °C until analysis.

### 2.3. Determination of Lignin Content

According to the method of Chen Xiao Guang et al. [28], 10 mg of the powder was accurately weighed and ground into a homogenate in a mortar with 95% ethanol. Then the homogenate was transferred to a 50-mL centrifuge tube. After centrifugation at 4000 rpm for 5 min, the pellet was washed twice with 5 mL of 95% ethanol and then washed twice with 6 mL of ethanol:n-hexane (1:2 (*V*/*V*)). The pellet was dried naturally, and 6 mL of 25% solution of acetyl bromide in glacial acetic acid was added to dissolve the precipitate, which was covered and sealed in a water bath at 70 °C for 30 min. Subsequently, 0.9 mL of 2 mol·L^−1^ NaOH, 5 mL of glacial acetic acid, 0.1 mL of 7.5 mol·L^−1^ hydroxylamine hydrochloride, and finally 15 mL of glacial acetic acid were added. Instead of the substrate, distilled water was used for the same reaction as a control. The absorbance of the supernatant was measured at 280 nm, and the absorbance of the dry sample per gram at 280 nm represented the lignin content per gram (OD·g^−1^DW) (OD means Optical Density, DW means dry weight). All the data were obtained from three biological replicates of each experiment.

### 2.4. qRT-PCR Analysis

Primer 5.0 was used to design RT-qPCR primers within conserved domain database sequence regions (Table 1) based on the nucleotide sequences of genes such as *PAL* (GenBank Accession No. KP100114), *C4H* (KP100113), *C3H* (KY078743), *4CL1* (MF346380), *4CL3* (MF346382), *F5H* (MG729630), *CCoAOMT* (AY651026), *CCR* (MG729629), *CAD* (KF758396), *COMT* (DQ665867), and *Actin I* (DQ665832). *UDP-GT* and *POD* were based on the ramie transcriptome [27], and had already been cloned by our research group.

The RT-qPCR reaction system contained 10 μL of ChamQ qPCR SYBR Green Master Mix (Vazyme, Nanjing, China), 2 μL of cDNA, 0.4 μL of Primer-P1, and 0.4 μL of Primer-P2. Distilled water was added to ensure a total volume of 20 μL. The amplification reaction program was 95 °C for 30 s, followed by 40 cycles of 95 °C for 10 s and 60 °C for 30 s (the annealing temperature was adjusted according to different primers). A total of three biological replicates were performed in the experiment, and the sum of all copies of the key genes in three periods and at the two sites was determined. Taking *Actin I* as the reference gene [29] and the xylem in the fast-growing period as a reference sample, the relative expression level of each gene was determined by analyzing data through the 2^−ΔΔCt^ method [30].

### 2.5. Heterologous Expression and Purification of Recombinant 4CL3

The Open Reading Frame (ORF) of *4CL3* was subcloned into the expression vector pQE30 vector (be donated by Xiangning Jiang from Beijing Forestry University), which contained multiple cloning sites of two restriction enzymes (*BamH*I and *Hind* III, TaKaRa, Beijing, China). The primers used to construct the 4CL3 protein expression vector are 4CL3- *BamH*I: CGCGGATCCATGGAGCGATCTGGGTTCGG and 4CL3- *Hind* III: CCCAAGCTTCGTCATATTTTGGAGCGAACTTTCT. The ligated vector was transformed into *Escherichia coli* M15 competent cells (be donated by Xiangning Jiang from Beijing Forestry University). Recombinant histidine-tagged proteins were purified using the His-Tag purification system (QIAGEN, New York, NY, USA). The purified proteins were used for further enzymatic assays.

### 2.6. Determination of Enzyme Activity

For the enzyme activity assay, the reaction system consisted of 50 μL of 20 mM ATP; 100 μL of 3 mM COA; 200 μL of 1 M Tris-HCl (pH 8.0) buffer; 300 μL of 17 mM MgCl_2_; 0.2 mM of the substrate, namely *p*-coumaric acid, cinnamic acid, caffeic acid, ferulic acid, and sinapinic acid, and 30 μg/mL purified recombinant protein. Sterile ddH_2_O was added to ensure a total volume of 1000 μL. The reaction system was incubated at 40 °C for 1 h. The reaction was terminated after boiling at 100 °C for 10 min, and the test was repeated three times. The reaction mixture was filtered through a 0.45 μm filter, and to detect changes in the substrates, 10 μL of the supernatant was used for HPLC analysis on a Waters 2695 C18 column (XDB-C18, 5 μm, Agilent, CA, USA). HPLC conditions were as follows: mobile phase: 1% H_3_PO_4_ aqueous solution (B phase) and acetonitrile (A phase), elution gradient: 5% A, 0–5 min; 5%–25% A, 5–35 min; 25%–95% A, 35–36 min; 95% A, 36–50 min; 95%–5% A, 50–51 min; and 5% A, 51–60 min.

### 2.7. Data Processing and Analysis

Data processing was performed using Microsoft Excel 2003 (Redmond, WA, USA). Statistical significance of the data and analysis of variance was performed and correlation coefficient was calculated using SPSS 20.0 software (version 20, SPSS Inc., Chicago, IL, USA). Multiple comparative analysis was performed on Turkey’s test. The histograms were generated and the Michaelis–Menten curve was fitted using Origin 9.0 software (*Origin*Lab, Northampton, MA, USA).

## 3. Results

### 3.1. Lignin Content in the Xylem and Phloem of Ramie Stems during the Three Periods

**Figure 2:**
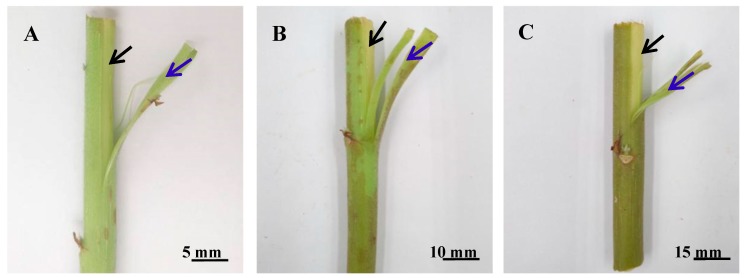
Morphology photographs of the xylem and phloem in ramie stems at three periods. (**A**, **B**, and **C**) Morphology photographs of xylem and phloem in ramie stems at the fast-growing period, maturation period, and late maturation period, respectively. Black arrow indicates the xylem section, and blue arrow indicates the phloem section.

To research the process of lignin development in ramie stems, the stems in the fast-growing period, maturation period, and late maturation period were selected. In the fast-growing period, the stem phloem appeared light green (Figure 2A). In the maturation period, the color of the stem phloem gradually deepened and became light brown (Figure 2B). In the late maturation period, the phloem and xylem of the stem became brown (Figure 2C). The results showed that the lignin content in the xylem and phloem of the stem gradually increased from the fast-growing period to the late mature period (Table 2). In the same period, the lignin content in the xylem was higher than that in the phloem. In the fast-growing period, the lignin content in the xylem was 5.7 times higher in the phloem. In the maturation period, the lignin content in the xylem was 5.3 times higher in the phloem. In the late maturation period, the lignin content in the xylem was 4.5 times higher in the phloem. Analysis of variance showed that the lignin content in the stem segments was significantly different between the xylem and phloem during the three periods (Table 3).

### 3.2. Expression Profile of Key Genes for Lignin Synthesis in the Stem

qRT-PCR analysis of 12 genes in the four pathways of lignin synthesis at two sites during the three periods.

**Figure 3:**
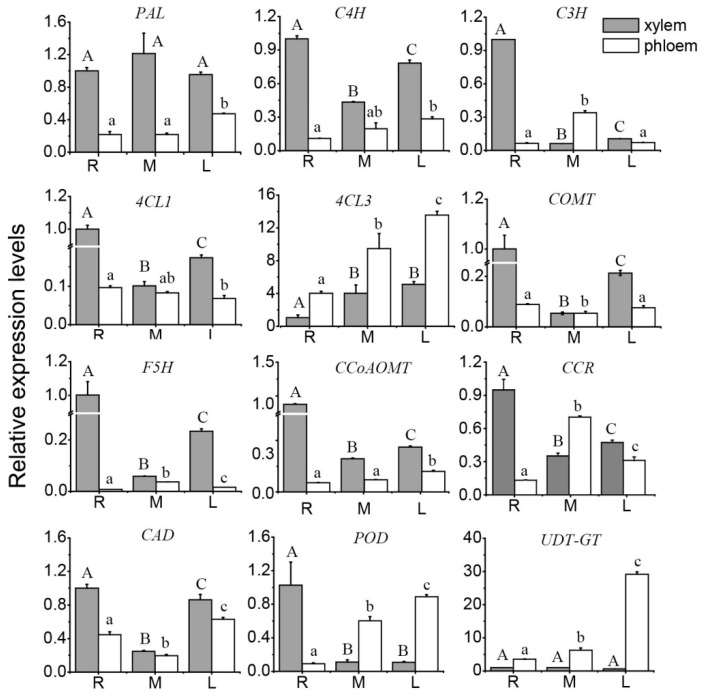
Relative expression levels of genes involved in the lignin biosynthesis pathway in the xylem and phloem of ramie at three periods. R: fast-growing period; M: maturation period; L: late maturation period; *PAL*: phenylalanine alanine ammonia-lyase; *C4H*: cinnamate-4-hydroxylase; *C3H*: coumarate-3-hydroxylase; *4CL*: 4-coumarate coenzyme A ligase; *COMT*: caffeic acid-O-methyltransferase; *CCoAOMT*: coenzyme-AO-methyltransferase; *F5H*: ferulate-5-hydroxylase; *CCR*: cinnamoyl-CoA reductase; *CAD*: cinnamyl alcohol dehydrogenase; *POD*: peroxidase; *UDP-GT*: coniferyle alcohol glucosyltransferase. Statistical significance of the data was performed by SPSS 20.0 software. Multiple comparative analysis was performed on Turkey’s test. Significant differences are represented by different letters.

In the phenylpropanoid metabolic pathway, we tested the expression level of five genes, including *PAL*, *C4H*, *C3H*, *4CL1*, and *4CL3*. The results showed that the relative expression levels of four genes (*PAL*, *C4H*, *C3H*, and *4CL1)* were higher in the xylem than in the phloem during the three periods, however, during the three periods, the relative expression of *4CL3* in the xylem was lower than that in the phloem, and during the maturation period, the relative expression level of *C3H* in the xylem was lower than that in the phloem. Furthermore, the relative expression of *4CL3* in the xylem and in the phloem was much higher than that in other genes (Figure 3).

In the lignin-specific synthetic pathway, we tested the expression of the three genes of *COMT*, *CCoAOMT*, and *F5H*. The result showed that the relative expression of the three genes of *COMT*, *CCoAOMT*, and *F5H* were higher in the xylem than in the phloem during the three periods. In the xylem, the relative expression levels of the three genes were the highest in the fast-growing period; in the phloem of the stem, the relative expression of *COMT* was the highest in the fast-growing period, the relative expression of *CCoAOMT* was the highest in the late maturation period, and the relative expression of *F5H* was the highest in the maturation period (Figure 3).

In the downstream lignin synthetic pathway, we tested the expression levels of *CCR*, *CAD*, and *POD*. The results showed that the relative expression levels of *CCR* and *CAD* were higher in the xylem than in the phloem during the three periods, except that the relative expression level of *CCR* was lower in the xylem than in the phloem in the maturation period. The *POD* relative expression level was higher in the xylem than in the phloem only in the fast-growing period. In the stem xylem, the relative expression levels of *CCR*, *CAD*, and *POD* were the highest in the fast-growing period; in the stem phloem, the relative expression of *CCR* was the highest in the maturation period, and the relative expression of *CAD* and *POD* was the highest in the late maturation period (Figure 3).

In the shunt pathway of lignin monomer synthesis, we tested the relative expression of *UDP-GT*, which was significantly higher in the phloem than in the xylem in the three periods. In the xylem of the stem, the relative expression of *UDP-GT* was not significantly different across the three periods. In the phloem of the stem, the relative expression of *UDP-GT* gradually increased with the growth of ramie and increased rapidly from the maturation period to the late maturation period. The highest relative expression level was reached in the late maturation period (Figure 3).

### 3.3. Correlation between the Lignin Content in Stem Segments and the Expression Level of Key Genes

Correlation analysis based on the lignin content and the CT value of genes expression (Table 4). During the fast-growing period of ramie stems, all p-values were between 0.000 and 0.002. So, lignin accumulation was significantly and positively correlated with the expression levels of the key genes in phenylpropane metabolic pathways, lignin-specific synthetic pathways, and downstream pathways of lignin synthesis. 

During the maturation period of ramie stems, *PAL*, *4CL1*, and *C4H* in the phenylpropanoid pathway, *F5H* and *CCoAOMT* in the lignin-specific synthetic pathway, and *CAD* in the downstream pathway of lignin synthesis all showed significantly positive correlations with lignin accumulation. A negative correlation with *C3H* in the phenylpropanoid metabolic pathway and *CCR* and *POD* in the downstream pathway of lignin synthesis was observed.

In the late maturation period, lignin accumulation was significantly positively correlated with the expression levels of the key genes in the phenylpropanoid pathway, the lignin-specific synthetic pathway, and in the downstream pathway of lignin synthesis, and all *p*-values were less than 0.005. Lignin accumulation had a significant negative correlation with the expression levels of *POD* in the downstream pathway of lignin synthesis, respectively.

In addition, the *p*-values of *UDP-GT* were 0.000 in the three periods. The *p*-values of *4CL3* were 0.01 in the late maturation period. Therefore the lignin accumulation in the three periods showed a significant negative correlation with the expression of *4CL3* and *UDP-GT*, respectively. 

### 3.4. Enzyme Assay of the Recombinant 4CL3 Protein of Ramie

**Figure 4:**
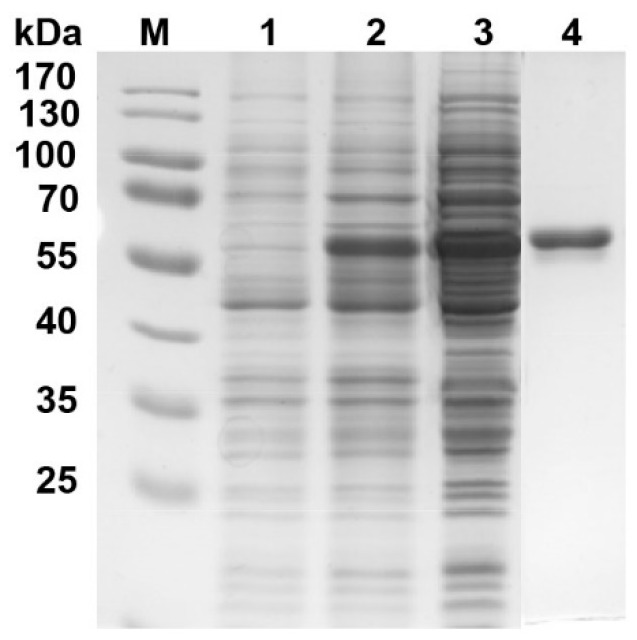
SDS-PAGE electrophoretic analysis of the 4CL3 recombinant protein of ramie. (M) protein marker; (1) total protein from strains with empty vector after IPTG (Isopropyl β-D-Thiogalactoside) induction; (2) total protein from treatment strains after IPTG induction; (3) protein in supernatant from treatment after IPTG induction; and (4) purified recombinant 4CL3 protein.

The aforementioned results showed that the expression of *4CL3* was negatively (to a significant extent) correlated with lignin accumulation (Table 4). The purpose of this study was to provide a theoretical basis for the use of genetic engineering to develop ramie plants that produce environmentally friendly ramie phloem fiber with a low lignin content. Therefore, the enzyme characteristic of 4CL3 from ramie was further researched. The open reading frame of *4CL3* was cloned from ramie and expressed in *Escherichia coli* M15. An enzyme assay was performed on the purified recombinant 4CL3 (r4CL3) protein (Figure 4) and r4CL3 catalytic presumed substrates, namely p-coumaric acid, cinnamic acid, caffeic acid, ferulic acid, and sinapinic acid were tested by HPLC.

According to the HPLC results, r4CL3 could catalyze three substrates (*p*-coumaric acid, cinnamic acid, and caffeic acid) to produce coenzyme A. However, r4CL3 showed no activity toward the substrates of ferulic acid and sinapinic acid. Enzyme kinetic analysis (Table 5) indicated that the catalytic efficiency of r4CL3 was the highest for cinnamic acid, followed by caffeic acid, and *P*-coumaric acid (Figure S1).

## 4. Discussion

### 4.1. Lignin Formation in the Xylem and Phloem of the Ramie Stem

In ramie stem tissues, primary fibers are attached to the phloem and secondary fibers are attached to the xylem. Fiber quality in the phloem is much higher than that in the xylem [31]. The cellulose and lignin content in the xylem and phloem of the ramie stem differed significantly at different developmental periods.

In our study, the lignin content in the xylem and phloem of the ramie stem during the fast-growing, maturation, and late maturation periods was determined, and the results showed that the lignin content gradually increased in the xylem and phloem of the ramie stem from the fast-growing period to the late maturation period. The lignin content in the xylem of the ramie stem was higher than that in the phloem during the same period; the lignin content in the ramie stem varied significantly at different sites and different developmental periods.

These results indicated that the lignification of ramie stems was continuously strengthened during maturation, and the degree of lignification in the xylem and phloem differed.

### 4.2. Correlation between Lignin Content in the Ramie Stem and the Key Genes for Lignin Synthesis

The study has shown that *CCOAOMT*, *4CL*, *COMT*, and *F5H* were the key genes for lignin metabolism in the xylem of flax [15]; *PAL*, *4CL*, *C4H*, *CCoAOMT*, *CAD*, and *CCR* were the key genes for lignin synthesis in the stem of common buckwheat, and the expressions of these genes were significantly and positively correlated with lignin content [16]. *PAL*, *4CL*, *F5H*, and *CCR* were the key genes for lignin metabolism in the rape stem, and the expression of these genes was positively correlated with lignin content [17].

Our study found that during the three periods, the lignin content in the ramie stem showed a significantly positive correlation with the expression of *PAL*, *C4H*, and *4CL1* in the phenylpropanoid pathway, with the expression of *CCoAOMT* and *F5H* in the lignin-specific synthetic pathway and with the expression of *CAD* in the downstream pathway of lignin synthesis. This result indicated that *PAL*, *C4H*, *4CL1*, *CCoAOMT*, *F5H*, and *CAD* are the key genes for lignin synthesis in the ramie stem.

The *POD* of kenaf was overexpressed in *A. thaliana*, which significantly increased the lignin content in *Arabidopsis* [32]. In the present study, the expression of *POD* in the downstream pathway of lignin synthesis was significantly and positively correlated with the lignin content in the fast-growing period and was significantly and negatively correlated with lignin content in the maturation and late maturation periods. This result indicated that *POD* exerted a positive regulatory effect on lignin synthesis in the ramie stem during the fast-growing period and a negative regulatory effect in the maturation and late maturation periods.

In this study, the expression of *UDP-GT* in the lignin monomer synthesis shunt pathway and *4CL3* in the phenylpropanoid pathway was significantly and negatively correlated with the lignin content in the three periods. This result speculated that *4CL3* and *UDP-GT* negatively regulate lignin synthesis in the ramie stem. The expression of *4CL3* and *UDP-GT* was negatively correlated with lignin accumulation in the ramie stem, suggesting that overexpression of *4CL3* and *UDP-GT* could facilitate lignin inhibition during ramie development. 

### 4.3. Enzyme Assay of the Recombinant 4CL3 Protein of Ramie

The *4CL3* recombinant protein of ramie showed a substrate preference for cinnamic acid during catalysis. However, cinnamic acid is not used as a substrate for *4CLs* in most plants, such as *Physcomitrella patens* [33]. The catalytic specificity of the ramie *4CL3* recombinant protein is similar to that of *Pa4CL1* in *Plagiochasma appendiculatum* [34]. It could only catalyze cinnamic acid, *p*-coumaric acid, and caffeic acid but could not catalyze ferulic acid and mustard acid.

The *4CL3* recombinant protein of ramie showed no catalytic activity for ferulic acid and sinapinonic acid, indicating that *4CL3* can only convert small-molecule substrates, especially cinnamic acid, p-coumaric acid, and caffeic acid. This is probably because its hydroxyl binding pocket is not large enough and it cannot accommodate larger substrates such as ferulic acid or sinapic acid. *At4CL2* variants with catalytic activity for ferulic acid and sinapinic acid in *A. thaliana* are produced by increasing the size of the substrate binding pocket [35].

In this study, the *4CL3* recombinant protein of ramie showed a substrate preference for cinnamic acid during catalysis. The expression of *4CL3* showed significant negative correlation with lignin accumulation in the three periods. Furthermore, the relative expression of *4CL3* was significantly higher than that of other genes in lignin synthesis pathway, and the relative expression of *4CL3* was much higher in the xylem than in the phloem. This study speculated that cinnamoyl-CoA catalyzed by 4CL3 may be mainly involved in the synthesis of ramie flavonoid compounds (Figure 1).

## 5. Conclusions

The critical period of lignin synthesis in the xylem of the ramie stem was the fast-growing period, and in the phloem, it was the late maturation stage. *PAL*, *C4H*, *4CL1*, *CCoAOMT*, *F5H*, and *CAD* were correlated with lignin accumulation in ramie. *4CL3* and *UDP-GT* were negative correlated with lignin accumulation in ramie. *4CL3* was involved in the synthesis of ramie flavonoid, and its overexpression of *4CL3* through genetic engineering would be expected to be an effective method of reducing the lignin content in the ramie stem.

## Figures and Tables

**Table 1:** Primers used in the experiment and its amplification products.

Gene	Primer Sequence (5′–3′)	PCR Amplicon Size (bp)	Tm (°C)
*PAL*	P1:GAGCAGCACAACCAAGACG P2:CCGTGGTCAGCACCTTCTT	202	49.2
*C4H*	P1:CATTCCTGCGAGGCTACTTG P2:CTGCTGAGCGTCAAGAATGTG	164	49.2
*C3H*	P1:GAGGTAAACGAGCAAGGACAAG P2:GAACAACCGCAACCAAGGA	105	58.9
*4CL1*	P1:AGAGGTCCCTGTTGCCTTTGTTG P2:TTTTGCCTGAAGGGGATTTAGTGA	152	60.0
*4CL3*	P1:ACCGCCGTGCTGCTGTATT P2:CAGCCCGAAGACGTGAAACA	171	61.8
*COMT*	P1:GACCAAGAACAACGACGGC P2:AATCTCGGATCTGTCCCATGG	171	60.0
*F5H*	P1:GTGCGAAGGTGAATGAGAGC P2:TGCACCTTCTTTAGATCCTCTG	175	60.0
*CCoAOMT*	P1:GGATGCTGACAAGGACAACT P2:GAGCCATTCCATAGGGTGT	102	49.2
*CCR*	P1:CCCGATGTTGTGGTTGATGAGTC P2:ACCAAATCCACGCCTTTCTCC	137	60.0
*CAD*	P1:GGCATGAGATTGTTGGAATTG P2:CATGGTTGAAGGTGTAAACGG	171	60.0
*POD*	P1:CCCAGGATAGCCATCAACAT P2:TTCCGACTCGTTTTCACCC	230	58.0
*UDP-GT*	P1:GGAGCAACAAGTCAACGCCT P2:CTCTTTTACCCTTTTCCTTATCGC	199	60.0
*Actin I*	P1:CGTTGAACCCTAAGGC P2:ATCCAGCACGATACCAG	137	reference gene

**Table 2:** Lignin content in the xylem and phloem of ramie stems at three periods (OD·g^−1^DW).

Tissue	Fast-Growing Period	Maturation Period	Late Maturation Period
Xylem	46.09 ± 3.091 cC	62.19 ± 1.013 bB	71.46 ± 3.669 aA
Phloem	8.04 ± 0.771 eD	11.74 ± 0.485 deD	15.95 ± 0.636 dD

Data are expressed as the mean of three replicates ± SD (*n* = 3). Different lowercase and uppercase letters indicate significant difference at *p* < 0.05 and *p* < 0.01, respectively.

**Table 3:** Analysis of variance of lignin content.

Variation Source	SS	DF	MS	*F*-Value
Tissue	10,369.440	1.000	10,369.440	368.635 **
Periods	840.654	2.000	420.327	14.943 **
Error	393.810	14.000	28.129	

SS: square sum; DF: degree of freedom; MS: mean square. ** indicate significant difference at *p* < 0.01.

**Table 4:** Correlation coefficients of lignin content and its related key genes expression.

Periods	*PAL*	*C4H*	*C3H*	*4CL1*	*4CL3*	*COMT*	*F5H*	*CCoAOMT*	*CCR*	*CAD*	*POD*	*UDP-GT*
Fast-growing period	0.983 **	0.981 **	0.986 **	0.981 **	−0.961 **	0.981 **	0.978 **	0.987 **	0.999 **	0.994 **	0.961 **	−0.987 **
Maturation period	0.953 *	0.972 **	−0.998 **	0.945 **	−0.916 *	0.025	0.985 **	0.997 **	−0.996 **	0.915 *	−0.990 **	−0.989 **
Late maturation period	0.998 **	0.997 **	0.934 **	0.992 **	−0.991 **	0.985 **	0.983 **	0.988 **	0.969 **	0.944 **	−0.988 **	−0.992 **

* and ** indicate significant differences at *p* < 0.05 and *p* < 0.01, respectively. *PAL*: phenylalanine alanine ammonia-lyase; *C4H*: cinnamate-4-hydroxylase; *C3H*: coumarate-3-hydroxylase; *4CL*: 4-coumarate coenzyme A ligase; *COMT*: caffeic acid-O-methyltransferase; *CCoAOMT*: coenzyme-AO-methyltransferase; *F5H*: ferulate-5-hydroxylase; *CCR*: cinnamoyl-CoA reductase; *CAD*: cinnamyl alcohol dehydrogenase; *POD*: peroxidase; *UDP-GT*: coniferyle alcohol glucosyltransferase.

**Table 5:** Kinetic analysis of r4CL3.

Substrate	Km (μM)	Vmax (nkat mg^−1^ Protein)	Kcat (s^−1^)	Kcat/Km (s^−1^·mM^−1^)
p-Coumaric acid	498.491	1.607	0.097	0.194
Cinnamic acid	449.926	4.842	0.291	0.248
Caffeic acid	194.426	0.746	0.045	0.231
Ferulic acid	ND	ND	ND	ND
Sinapic acid	ND	ND	ND	ND

ND, no detectable activity.

## Supplementary Materials

The following are available online at https://www.mdpi.com/2073-4425/10/5/389/s1, Figure S1: HPLC chromatogram of reaction products generated from the recombinant 4CL3 protein catalysis of different substrates.
