# Supplementary material for: Correlation Analysis of Lignin Accumulation and Expression of Key Genes Involved in Lignin Biosynthesis of Ramie (Boehmeria nivea)
Source: Genes (Basel). 2019 May 22;10(5):389. doi: 10.3390/genes10050389 (PMC6562848; doi:10.3390/genes10050389)
Supplement: Supplementary file 1 [file genes-10-00389-s001.pdf]

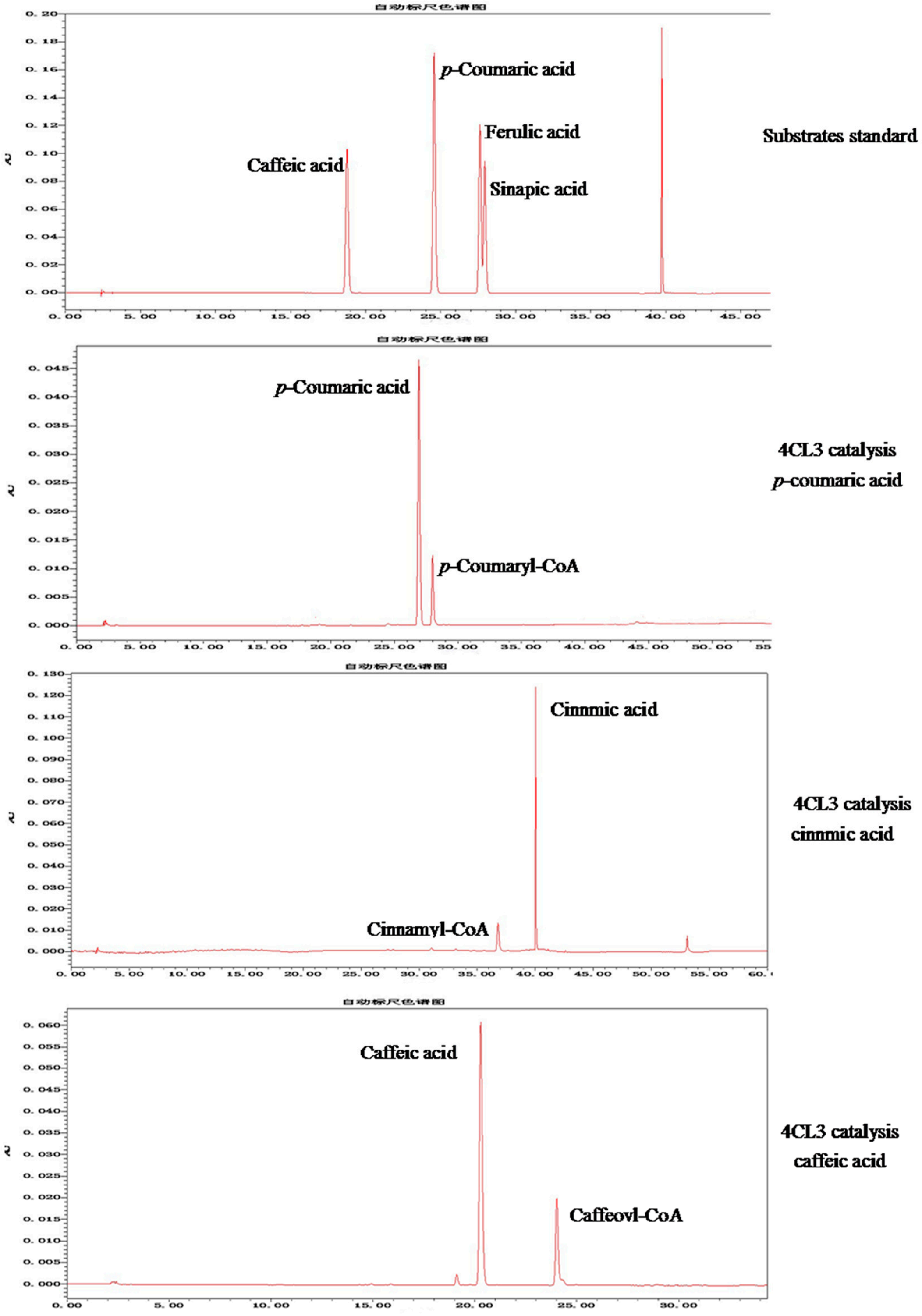

Figure S1. HPLC chromatogram of reaction products generated from the recombinant 4CL3 protein catalysis of different substrates.
